# Fibrolipomatous hamartoma of the median nerve: An unusual cause of carpal tunnel syndrome

**DOI:** 10.1002/ccr3.7022

**Published:** 2023-03-02

**Authors:** Mario Gilberto Siqueira, Roberto Sérgio Martins, Luciano Foroni, Adilson J. M. de Oliveira, Gustavo Lordelo, Carlos Otto Heise

**Affiliations:** ^1^ Peripheral Nerves Group Hospital das Clinicas da Faculdade de Medicina da Universidade de São Paulo São Paulo Brazil; ^2^ Neuroscience Center Clinica Girassol Luanda Angola

**Keywords:** hamartoma, median neuropathy, median neuropathy, peripheral nerve tumor

## Abstract

Fibrolipomatous hamartoma is a rare benign tumor‐like condition that affects most commonly the median nerve. The diagnosis is usually confirmed through its typical appearance on magnetic resonance imaging (MRI) without the need for a nerve biopsy. There are divergent views regarding treatment of this entity, but open carpal tunnel release for nerve decompression currently constitutes the standard care for alleviation of compressive neuropathy of the median nerve. In this report, we describe a case of fibrolipomatous hamartoma that was diagnosed via MRI and underwent open carpal tunnel release, with alleviation of the patient's symptoms.

## 1 INTRODUCTION

Fibrolipomatous hamartoma (FLH) was first described by Mason in 1953.^1^ It is a rare, benign tumor‐like lesion of peripheral nerves for which the etiology is unclear. It is characterized by excessive proliferation of fibroadipose tissue, which infiltrates the epineural and perineural elements of the nerve. While this pathological condition can occur anywhere in the body, the most common site according to the literature is the median nerve.^2^ It has been considered congenital in origin and has been commonly associated with macrodactyly.^2^ The cause and the best treatment for FLH have not yet been clearly defined. We report a case of secondary carpal tunnel syndrome due to FLH on the median nerve, which was treated surgically.

## 2 CASE REPORT

**FIGURE 1:**
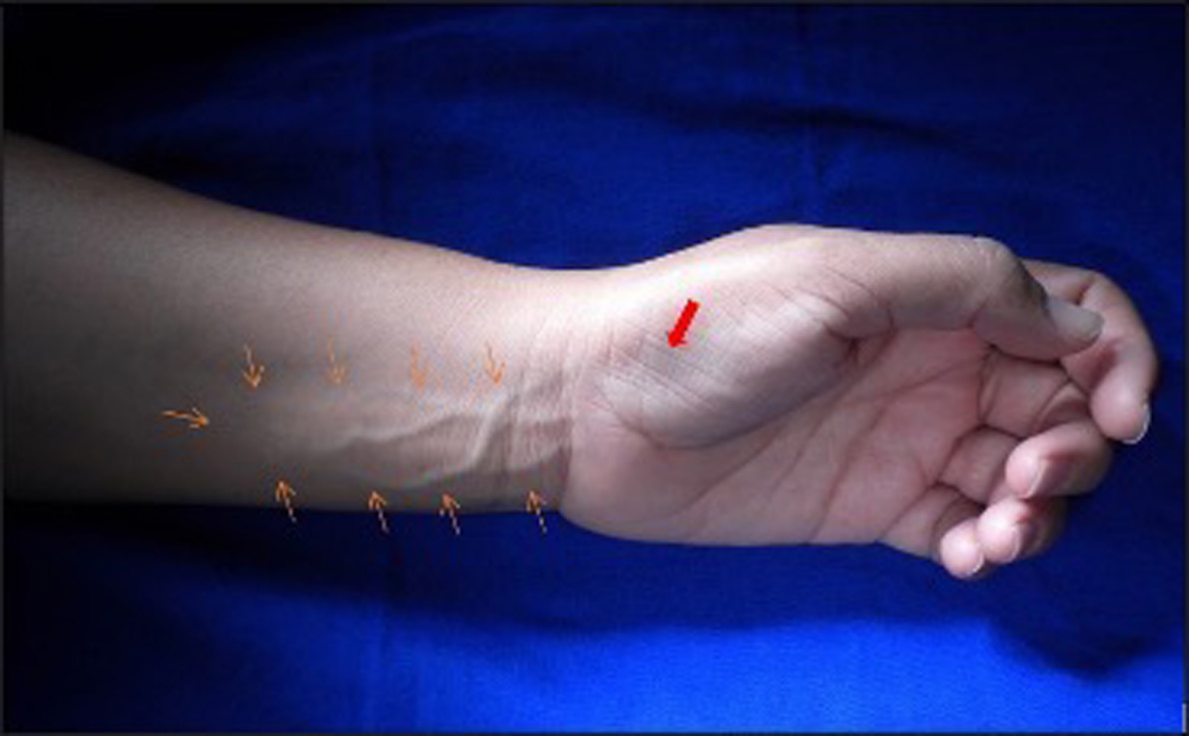
Large mass in the volar aspect of the distal forearm (orange arrows). Note the partial atrophy of the thenar muscles (red arrow).

A 46‐year‐old left‐hand‐dominant female patient presented to the hospital's outpatient clinic with a lump in the left distal forearm. The mass had slowly and progressively grown since adolescence, and over the last 4–5 years, some weakness of the hand had appeared. She complained of some loss of sensitivity of the thumb, index finger, and middle finger, and also of a shock‐like sensation when the mass was tapped. A physical examination revealed a soft palpable mass in the volar aspect of the left distal forearm. The shapes and sizes of the fingers were all normal. Hypoesthesia of the fingers was observed, along with atrophy of the thenar muscles. Tinel's sign was positive with percussion over the mass and over the carpal tunnel (Figure 1).

**FIGURE 2:**
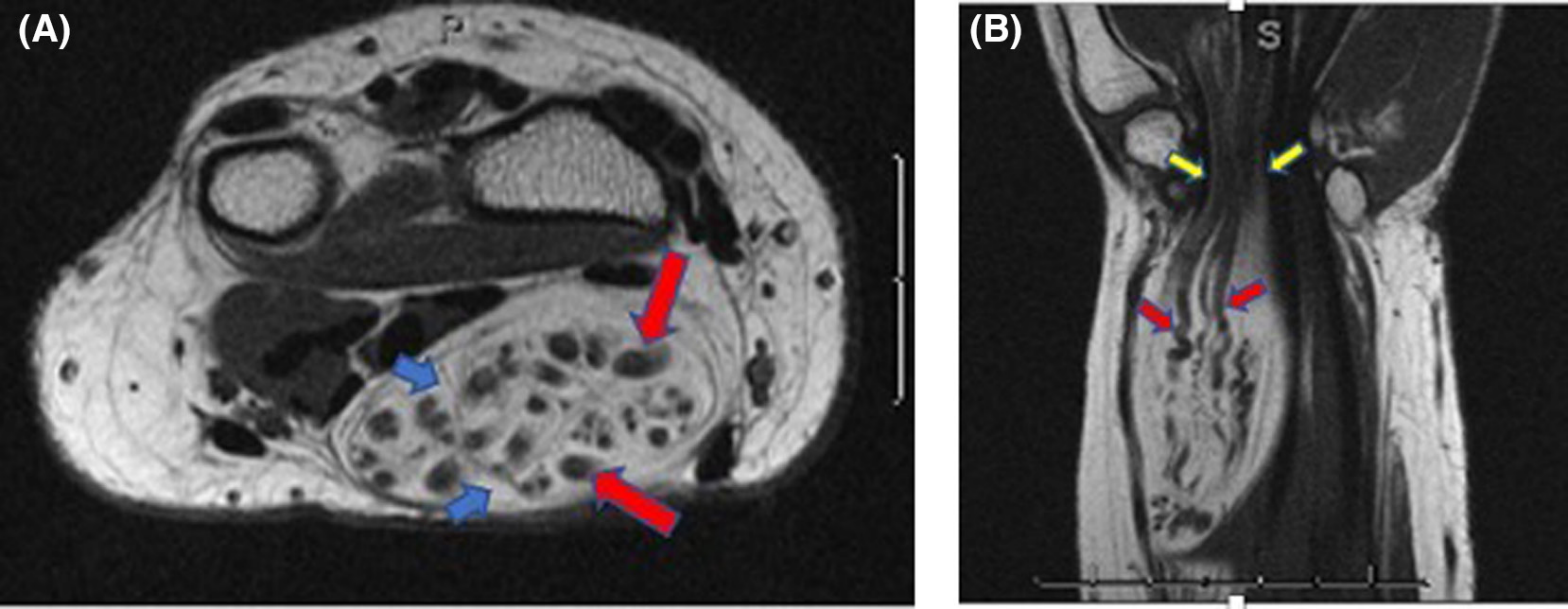
Magnetic resonance imaging of the fibrolipomatous hamartoma in T1‐weighted images showing typical findings. (A), Axial image demonstrating marked enlargement of the median nerve. Low‐intensity structures (nerve fascicles) (red arrows) are surrounded by high signal intensity (fat) (blue arrows) resembling a “co‐axial cable‐like appearance,” for the nerve in the distal forearm. (B), Coronal image demonstrating the median nerve fascicles (red arrows) inside the same large mass of the distal forearm, as serpiginous low‐intensity structures surrounded by fat, with “spaghetti‐like appearance.” Note the marked constriction of the mass inside the carpal tunnel (yellow arrows).

Coronal magnetic resonance imaging (MRI) revealed fusiform swelling of the median nerve, from the distal forearm to the proximal palm, with a length of 13 centimeters and a diameter of 5 cm at its widest point, with evident compression of the nerve by the transverse carpal ligament. In axial imaging, the mass was shown to have an oval shape, and T1‐weighted imaging had a typical “co‐axial cable‐like” appearance due to a low‐intensity nerve fascicle within the high‐intensity adipose tissue. In the coronal imaging, the mass also had a typical “spaghetti‐like” appearance, and evident compression of the mass in the carpal tunnel was demonstrated (Figure 2A,B).

**FIGURE 3:**
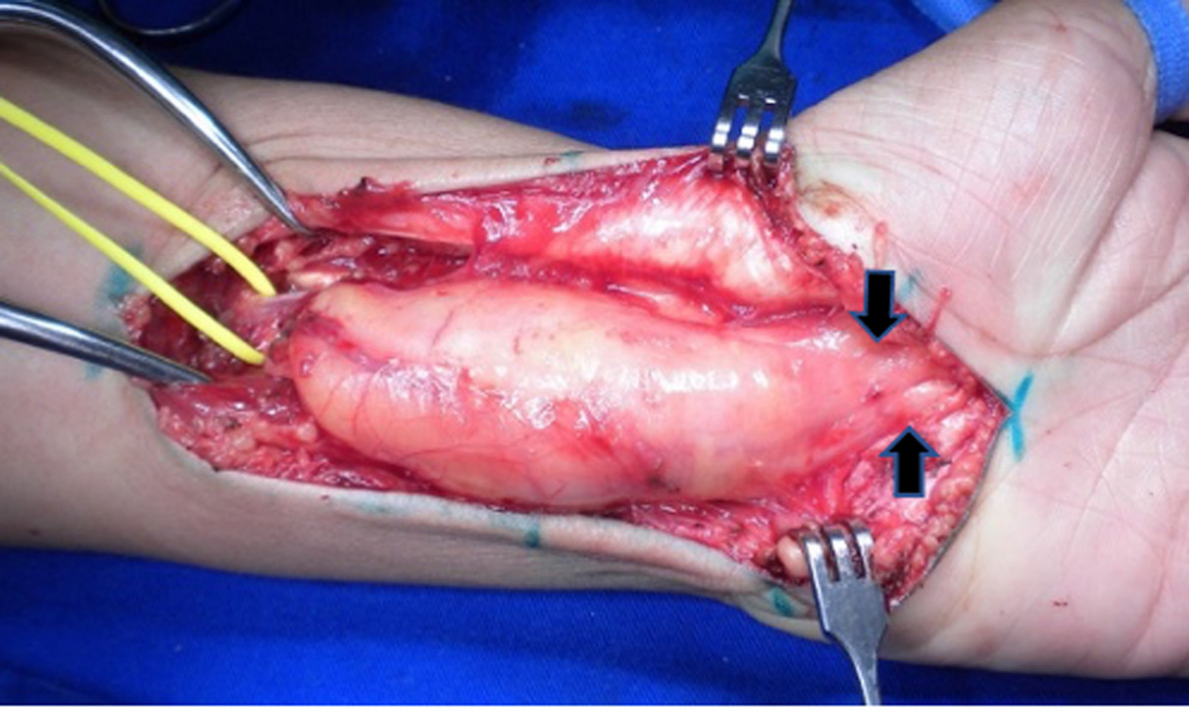
Intraoperative photograph showing the enlarged median nerve with fibroadipose tissue proliferation. Yellow vessel loop encircling the proximal median nerve. The enlarged nerve was decompressed from the distal forearm to the palmar region. Note the area of compression of the mass inside the opened carpal tunnel (black arrows).

The symptomatic compression of the mass by the carpal ligament formed the indication for surgical treatment. A longitudinal incision was made alongside the mass in the volar aspect of the distal forearm and entering the palmar area. After dissection of the subcutaneous tissues and opening of the fascia of the forearm, a yellow sausage‐like mass was exposed, without infiltrations into these surrounding tissues. The transverse carpal ligament was found to be severely compressing the mass in the carpal tunnel. It was carefully released (Figure 3). Biopsy of the tumor was not done.

The hypoesthesia and the Tinel sign disappeared after the operation, but the atrophy persisted. Soon after the surgery, the patient started to complain of intermittent burning pain at the surgery site, which disappeared spontaneously in a few weeks. Over a follow‐up period of 6 years, the mass did not increase in size and, except for the mild weakness in the hand, the patient is asymptomatic.

## 3 DISCUSSION

Fibrolipomatous hamartoma is also known as neural fibrolipoma, lipofibroma, fatty infiltration, intraneural lipoma, fibrofatty proliferation, and lipofibromatous hamartoma. All of these were grouped under the term “lipomatosis of the nerve” by the World Health Organization in 2002.^3^ The inconsistency in terminology causes confusion among physicians.^3^, ^4^

FLH is a rare occurrence. In a series of 146 peripheral non‐neural sheath nerve tumor cases collected by David Kline over a 30‐year period, only four cases of FLH were found.^5^ According to a review of the literature by Marek et al,^6^ 401 papers about this disease had been published, from the first description in 1953 up to November 2018. Over that 65‐year period, these papers encompassed 1025 cases, that is, an average of 15.7 cases published per year.

The disease is believed to be of congenital origin, is almost always described in the first three decades of life, and is more common among women (2:1).^7^ Our report came from a woman who was somewhat older (46 years of age) than most previous cases.

The most common presentation is, like in our case, a mass or swelling on the volar aspect of the wrist or distal forearm,^7^, ^8^ usually involving the median nerve (almost 60% of cases).^6^

An association of FLH with nerve territory overgrowth is present in about 60% of the cases, and the mass is always distal to the affected nerve, sometimes producing bone overgrowth and macrodactyly, with lipomatous macrodystrophy of muscles and subcutaneous fat in the region supplied by the nerve.^9^ Our patient had none of the abovementioned overgrowths.

The disease history is usually long. In our case, it was more than 30 years.

The patients may complain of increasing pain, tenderness, diminished sensation, paresthesia, and loss of muscle strength in the median nerve territory, associated with the gradually increasing mass, which causes compression neuropathy. As the median nerve is frequently involved, carpal tunnel syndrome is a frequent late complication of these lesions.^10^ Nonetheless, despite the intense compression of the lesion at the carpal tunnel in our case, the patient only had a few complaints.

The imaging features seen on MRI highlight the distinction between the abundant fibrous tissue around the fascicles and the adipose tissue within the epineurium. This arrangement results in massive nerve enlargement with a pathognomonic MRI appearance in axial images, often referred to as “co‐axial cable‐like appearance.” In the coronal plane, the thickened nerve bundles surrounded by uniformly distributed fat appear as serpentine, low‐intensity structures encircled by high‐intensity signal (fat), giving rise to a “spaghetti‐like appearance”.^11^ These findings are accepted as pathognomonic of Lipofibromatous hamartoma.^11^

If the MRI studies support the diagnosis of FLH, pre‐ or operative biopsy of the lesion should be avoided. The biopsy will not reveal any new diagnostic features and poses a small but significant risk of permanent neurological deficits.^5^ In our case, the MRI findings were typical and a biopsy could be avoided.

Lipofibromatous hamartoma is considered to be a hamartoma because of the overgrowth of normal connective tissue components: fat and fibrous tissue. On microscopic examination, a fibrofatty expansion of the epineural space is seen, with splaying of the nerve bundles. Perineural and endoneurial fibrosis may also be seen.^7^

The treatment options for FLH include a wide range of options, from close observation to complete nerve resection. Close observation should be advocated in the absence of neurological symptoms.^12^ As the median nerve is the one that is most affected by the disease, it commonly becomes entrapped at the carpal tunnel due to enlargement of the nerve. Consequently, the most frequent surgery consists of carpal tunnel release.^2^ The results from nerve decompression are generally good, although reoperation may be necessary since FLH is frequently a progressively enlarging lesion.^13^ Intraneural dissection has also been reported by some authors as a means for reducing nerve volume. This procedure consists of meticulous dissection of the fat from within the nerve fascicles,^14^ but extensive microsurgical intraneural dissection can lead to significant ischemic complications. The risk of permanent neurological deficit outweighs the potential benefits of this treatment.^4^ Complete tumor excision, with or without reconstruction of the nerve with grafts, is only rarely recommended. Our case was treated through simple decompression of the carpal tunnel, by sectioning the transverse carpal ligament. The patient has been asymptomatic for 6 years.

## 4 CONCLUSION

Fibrolipomatous hamartoma is a benign condition that occurs predominantly in the median nerve. There are divergent views regarding treatments for this condition and some cases that are very slow‐growing and do not have any significant neurological manifestations can be managed conservatively. The lesion can be debulked, but extensive intraneural dissection can lead to ischemic complications. Complete excision of the mass with repair of the nerve is advocated by some authors, but this can result in important neurological loss. In our opinion, the best treatment for carpal tunnel syndrome originating from a fibrolipomatous hamartoma is fasciotomy of the distal forearm, neurolysis of the mass and sectioning of the transverse palmar ligament to decompress the tumor. Owing to the progressive nature of this disorder, repeated procedures may be necessary.

## AUTHOR CONTRIBUTIONS

**Mario Gilberto Siqueira:** Conceptualization; data curation; formal analysis; investigation. **Roberto Sergio Martins:** Conceptualization; data curation; formal analysis; investigation. **Luciano Foroni:** Conceptualization; investigation; validation. **Gustavo Correa Lordelo:** Conceptualization; data curation; methodology; writing – original draft. **Carlos Otto Heise:** Supervision; validation; writing – review and editing.

## FUNDING INFORMATION

None.

## CONFLICT OF INTEREST STATEMENT

The authors declare that they have no conflict of interest related to this publication. This work did not receive any funding.

## PATIENT CONSENT STATEMENT

Written informed consent was obtained from the patient to publish this report in accordance with the journal's patient consent policy and it and is in the possession of the authors.

## Data Availability

Data related of this paper are available for consultation if requested.
